# Supplementary material for: Patterns of Intron Gain and Loss in Fungi
Source: PLoS Biol. 2004 Nov 30;2(12):e422. doi: 10.1371/journal.pbio.0020422 (PMC532390; doi:10.1371/journal.pbio.0020422)
Supplement: Table S1 — Also available at http://genes.mit.edu/NielsenEtAl/. (4.3 MB ZIP). [file pbio.0020422.st001.zip › NielsenEtAl/html/838.html]

AN8800.1.NCU05642.1.MG04872.1.FG05963.1


```
 CLUSTAL W (1.82) Multiple Sequence Alignments - Introns Inserted


Sequence 1: NCU05642.1	778 aa
Sequence 2: FG05963.1	800 aa
Sequence 3: MG04872.1	803 aa
Sequence 4: AN8800.1	838 aa
Alignment Length: 891 aa
Number Identitical Residues: 268 aa
Alignment Score (without introns) 15690


MG04872.1 	--------------------------------MASP-------------------TIKVH
NCU05642.1	--------------------------------MAA---------------------ITVH
FG05963.1 	--------------------------------MES---------------------LKLY
AN8800.1  	MPLLPLRHLRLLSSLRSPRPVLFSQLSVLARFMATAREQPAWRQPTAHPEAQSLPSLKVY
          	 .  .       ::  :. .   :. :  :  * :. ...:  ..:: ..:.: .::.::

MG04872.1 	NSLQPGGPVPFEPKEKG--NVSWYACGPTV~YDQSHLGHARNYVSTDIIRRIFLHYFDYK
NCU05642.1	STLKPGPPVPFVPKEEG--AISWYACGPTV0YDLSHLGHARNYVSTDIIRRILMHYFDYK
FG05963.1 	NSLKPGAPVPFVPIEKG--KVSWYACGPTV~YDKSHLGHARNYVSTDIIRRILMHYFGFE
AN8800.1  	NSLTKS-KTPFIPIDPHGKKVTWYACGPTV~YDDAHLGHARNYVSTDIIRRIMRDYFKFN
          	.:*  .  .** * :  .. ::******** ** :*****************: .** ::

MG04872.1 	VKFV~MNITDIDDK~I-----IIRARRQRLL-DLEKKKAYTPEELHALGAKAFLAYAQSN
NCU05642.1	VNFV0MNYTDVDDK0I-----IIKARRKRLL-ELEKEKPYTPEQIHNLIFKAFQAYTKSL
FG05963.1 	VKFV~MNITDIDDK0I-----IIKARRQRLL-ELEKNKNYSKDELRDLALAAFQAYAKSS
AN8800.1  	VHFV~MNITDVDDK0VNGVQIILRARQQHLFTKFVTAHPTVDEEVLDVAKKAYAAYLKKN
          	*:** ** **:*** :.. . *::**:::*::.: . :    :::  :   *: ** :. 

MG04872.1 	LPLLV-EAEALDEKNYEQRRDKAYGGVLAGGALTADGKPGDGEAKIKMHLSNMDAAAKGL
NCU05642.1	PLLAPQDAGPLDEKNYEERKQAAYGVVLAGGPLVPETKPGDAEAKVKMHLNNMDAAAEAL
FG05963.1 	LPLLLKDGEDIDATNYPQRREAGYGHVLAGGTISGEGKPGDDEAKVKMHLSNMTSAAEAI
AN8800.1  	LPLLSPELPPSD---YSKEVEKAYAVILNGGPLPGNEKAGDDEAKIKMHIKTVSSAANVI
          	  *   :    *   * :. : .*. :* **.:  : *.** ***:***:..: :**: :

MG04872.1 	RNGKVFGS-------------ADEVLLPYLDSLYKETIDTSDQSMFTDLTRRMEADFFDD
NCU05642.1	KNGSGLDA-------------AEDVLLPYLDSLYKETIDTSDQTIFTDLTKTMEKAFADD
FG05963.1 	ASGEIFPG-------------TDEILLPYLDSLYKETIDTRDQTMFTDLTQSMEKLFTDD
AN8800.1  	VEAEKLDKSAQGTFPERFYTDAQDLLLPYLDALEGSLIDADDHSIFTKLTRKYEERFMKD
          	 ... :  ::..: ..   :.::::******:*  . **: *:::**.**:  *  * .*

MG04872.1 	MDNLNNLRPDEITRVTEYVPQIADFVDRIVKKGFAYE-SEGSVYFDIAAFEKGGNTYARL
NCU05642.1	MEALNVLPPDVITRVTEYVPQIVAFVEQIIQKGFAYE-ADGSVYFDIGAFEKAGNTYARL
FG05963.1 	MDALNVLRPDVITRVTEYVPQIADFVKTIVDKGFAYE-ADGSVYFDITAFEKAGNTYARL
AN8800.1  	MDDLNVLRPDELTRVTEYGQEIADFVEKIVENKFGYVSADGSVYFDINAFEAAGFPYARL
          	*: ** * ** :******  :*. **. *:.: *.* :::******* *** .* .****

MG04872.1 	KPESRGDKALQEEGEGSLSKGAGGKRSPGDFALWKQSKPGEPYWPSPWGNGRPGWHIE~C
NCU05642.1	RPESKNDTALQEEGEGSLSTALGGKKRSGDFALWKKSKPGEPYWPSPWGAGRPGWHIE~C
FG05963.1 	RPDNRNDKSLQEEGEGSLSKGLSGKKNPGDFALWKKSKAGEPFWSSPWGDGRPGWHIE2C
AN8800.1  	EPWSRSDNKLLAEGEGSLANKTTEKRSKSDFALWKSSKPGEPSWSSSWGRGRPGWHIE~C
          	.* .:.*. *  ******:.    *:  .******.**.*** *.*.** ******** *

MG04872.1 	S~VMASDRLGAQMDVHSGGIDLAFPHHDNELAQSEAYYCEHGKGEHTWVKYFL~HMGHLS
NCU05642.1	S0----------------------------LAQSEAYFHDCCKGEHTWVKYFL0HMGHLS
FG05963.1 	S~VMAGAVLGSNMDIHSGGIDLAFPHHDNELAQSEAYFCEHGKGEHTWVNYFL~HMGHLS
AN8800.1  	S~AMASARLGKHMDIHSGGIDLAFPHHDNELAQSEAYWHSHG---DQWVNYFL~HMGHLS
          	*   :.   .   .  :.. . : .  ...*******: .     . **:*** ******

MG04872.1 	ISGSKMSKSLKNFQTIKDALATT-YSARNLRIVFMMGRWNEGVEISPELRVQADSWENTV
NCU05642.1	IAGSKMSKSLKNFQTIQDALAST-YTARNMRIVFLLSRWNDGVEISPDMRKQADNWETTL
FG05963.1 	ISGSKMSKSLKNFQTIQDALATT-YSSRGMRIVFLMGRWNDGVEISPDMRLQADNWESTI
AN8800.1  	IQGSKMSKSLKNFTTIREALERKEWTPRSLRIVFLLGGWKDGIEITDELVSAGNSWEDKL
          	* *********** **::**  ..::.*.:****::. *::*:**: ::   .:.** .:

MG04872.1 	N0QFFTNTKSWLAEAGVASTNDGLESLSISDDAA-QQGLLADLDQAKKDTQAFLTNSFDT
NCU05642.1	D~NFFTNIKARLSEVDASS---GIKDLSLSEDAG-ARGLLEDFEQAKKDLDAALRNSFDT
FG05963.1 	S0NFFINVKALLAEAGITQ---GVKSLSLSANGKSSEGLLGELEQAKQEFEGAMTNSFDT
AN8800.1  	N~NFFIRVKDPAARQGPAS----------STDTT----LAEALEAAKSAVHEQLCDSFNT
          	. :** . *   :. . :.          * :      *   :: **.  .  : :**:*

MG04872.1 	NGALRTIAELVNKVNVHKREHKNDADLVAIERIARWVTRMVGIFGLDANAKPPYNGLGWV
NCU05642.1	PTAMQVLLKLVRNANIYMNSS--NPNVQALESVARWVTKIVGVFGLDASAQPPYDGLGWS
FG05963.1 	PKAMSVILKLVNTANVHLRDNK-DADLVALESIARWITKIVGIFGLDSNASPPYEGLGWA
AN8800.1  	PGMMNSISELITKYNIADKST---LNPKDVEAVARWVTYMVNVLGLNGQTPADSREIGWS
          	   :  : :*: . *:  ..     :   :* :***:* :*.::**:..: .    :** 

MG04872.1 	SDPSAG---LDLEAVRKTYASVRDEIKTSG------FAESVIAVLDTQSPASDVQKLEDS
NCU05642.1	SATASNKANVDPQTAVKPYAAVFNQVKQEVAALDLSGNDSIKSLLEQQNPDGEFAELEKS
FG05963.1 	TTIAS---DVEPKTAVQPYADAFAKVKSDVSGLSLESGE-ISSLLEQ-NPTSEFESIAAG
AN8800.1  	GIDVP----EEAKPFLYPLSAMRDALRQSARAKTLNAGEISKIVEQEAIPEATTEKTK--
          	    .     : :.   . :     :: .  .   .  :    : :   * .   .    

MG04872.1 	GVTDPERLAYPFVDAVAKLRDELRRTVSS--AASPEQKKAILSLSDRIRDHDLTNLGVYL
NCU05642.1	GEKDAEKLALPFIRATSRLRDELRATISSGVALDPKVKQAILALSDRIRDYDLTDLGVQL
FG05963.1 	GSRDPEQLAMPYLRAVSKLRDELRSIVGN---QTPDTKKAILALTDRIRDDDLTNLGVYL
AN8800.1  	----------PYATVVSNFRTKVSSLQDS-----GDLGKEVLALCDRLRDVDLFDLGVYL
          	          *:  ..:.:* ::    ..      .  : :*:* **:** ** :*** *

MG04872.1 	DDRPDGQPSLIKFVPAAQLIAAREEKAAQQAAKERAKEEA~RLEKERKEQERWAKAAVKP
NCU05642.1	DDRVDG-PSLVKFVPASKLIQAREEKAALLAEKARQKEEA1RKAKEKAEQEKWEKAKLSP
FG05963.1 	DDRPDGQASLIKFIPAAELIAAREEKVAQAAEKARKKEEA~RLAREKADQEAREKAKVRP
AN8800.1  	EDR-ENLPAIVRPVSR-DLIQAREEKAAQALQKQRERE--~--AKEKEALKKLEKGKLSH
          	:** :. .:::: :.  .** *****.*    * * :*      :*:   :   *. :  

MG04872.1 	QDMYRSETEKYSEFDDEGIPTKMKDGSEVTKSAAKKLKKDWEKQKKLHEEYLKKFGGS-
NCU05642.1	QDMFKSD-AKYTEFDADGLPTKLADGNEVPKSQVKKLKKEWEKQKKLHEEYLAKFASA-
FG05963.1 	EDMFKGD-ERYSAWDEQGMPTKMKDGTDVPKSQLKGLKKQWDRQKKAHDDLKAKGLL--
AN8800.1  	LEMFRTN--EYSAWDEEGIPTRDAAGEEIAKSRSKKLRKDWERQKKLHEAWLASQQGSK
          	 :*:: :  .*: :* :*:**:   * ::.**  * *:*:*::*** *:    .   :.
```
